# Medial Calcar Support and Radiographic Outcomes of Plate Fixation for Proximal Humeral Fractures

**DOI:** 10.1155/2015/170283

**Published:** 2015-01-27

**Authors:** Shih-Jie Lin, Yao-Hung Tsai, Tien-Yu Yang, Shih-Hsun Shen, Kuo-Chin Huang, Mel S. Lee

**Affiliations:** ^1^Department of Orthopaedic Surgery, Chang Gung Memorial Hospital, Chiayi City, Taiwan; ^2^College of Medicine, Chang Gung University, Taoyuan City, Taiwan; ^3^Graduate Institute of Clinical Medical Science, Chang Gung University, Taoyuan City, Taiwan

## Abstract

Plate fixation remains one of the most popular surgical procedures for treating proximal humeral fractures (PHFx); however, substantial rates of complications have been reported in the literature. The objectives of the study were to examine how medial calcar support (MCS) affects the radiographic outcomes and to determine the prognostic factors predicting treatment failure. We performed a retrospective cohort study of 89 adult patients who had PHFx and were treated with plate fixation at our institution in 2007–2011. The enrolled patients were separated into two groups according to disruption of medial calcar. Our results revealed an increased rate of poor radiographic outcomes in patients with disrupted medial calcar. Osteonecrosis of the humeral head and redisplacement were the two radiographic outcomes which had a positive causality with disruption of medial calcar (*P* = 0.008 and 0.050, resp.). Deficient medial calcar, inadequate reduction, diabetes mellitus, chronic kidney disease, and chronic liver disease were all significant predictors for the development of osteonecrosis in patients after PHFx surgery. Inadequate reduction was also a predictor for redisplacement. We confirmed that the restoration of medial calcar as well as comorbid conditions plays key roles in treatment of patients having PHFx with disrupted medial calcar.

## 1. Introduction

Regarding the complex fractures of proximal humerus (PHFx), there is no consensus on the optimal treatment [1]. Overall, open reduction and internal fixation with plates and screws, which may restore the anatomy of proximal humerus, have yielded satisfactory results and remained one of the most popular surgical procedures for treating theses fractures [1]. Biomechanical studies have shown that the medial calcar support (MCS) plays a key role in PHFx surgery, offering mechanical stability during and after reduction and thus preventing complications such as nonunion, malunion, and/or loss of reduction [2–4]. Hertel et al. [5] reported in his clinical study that the degrees of MCS deficiency were the most important predictors of humeral head ischemia, affecting perfusion of the humeral head by the vessels in the posteromedial periosteum and thus increasing complications such as osteonecrosis of the humeral head. The MCS consists of two main components: the length of the posteromedial metaphyseal extension and the integrity of the medial hinge [2–5]. To the best of our knowledge, this is the first to study on the radiographic outcomes of plate fixation for PHFx in patients with MCS deficiency. The information from this study may be valuable in improving plate fixation for PHFx, thereby reducing the complications such as loss of fixation or osteonecrosis of the humeral head.

Our hypothesis was that the presence of MCS deficiency would have a higher rate of poor radiographic outcomes in patients after plate fixation for their PHFx. Through a retrospective cohort study design, the purpose of this study was (1) to determine the causal relationship between the presence of MCS deficiency and the poor radiographic outcomes and (2) to explore the prognostic factors predicting treatment failure (poor radiographic outcomes).

## 2. Patients and Methods

### 2.1. Study Subjects and Grouping Criteria

**Figure 1:**
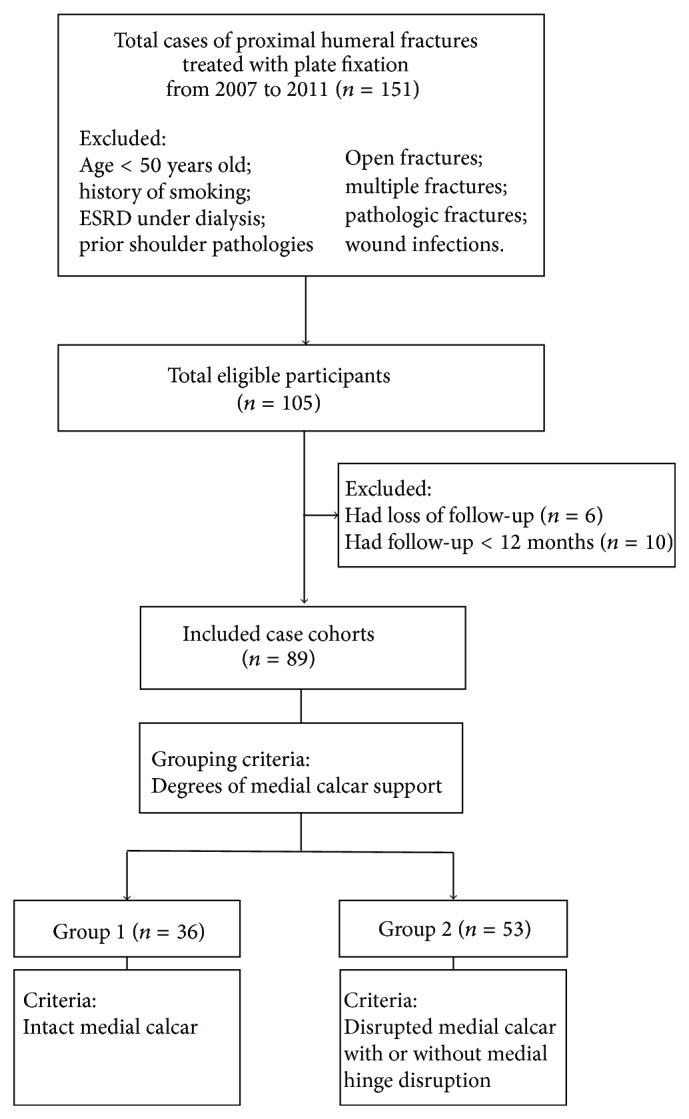
The flowchart of patient selection process.

This retrospective cohort study included all patients diagnosed with PHFx and treated with plate fixation at our institution between January 2007 and December 2011. After obtaining approval from the Institutional Review Board (IRB), cases were identified by matching the International Classification Diseases, 9th Revision, Clinical Modification (ICD-9-CM) codes specific for PHFx (812.00–812.19) in the computerized registry database of the hospital. We excluded patients aged < 50 years and those with open or multiple fractures, underlying bone pathologies and medical problems such as end-stage renal disease (ESRD) under dialysis, malignancies, deep infection, and smoking. The medical records were reviewed and confirmed by two independent researchers (SJL and KCH). Of the 105 eligible patients, 16 were further excluded from the study owning to inadequate (<12 months) or loss of follow-up. Final included case cohorts involved 89 patients (18 men, 71 women; 89 shoulders), who were followed for a minimum of 12 months (mean/median, 27/22 months; range 12–67 months) (Figure 1).

All these cases were treated with two kinds of plate: Proximal Humerus Internal Locking System, PHILOS (Synthes, Oberdorf, Switzerland) and conventional cloverleaf plate (Synthes, Paoli, PA). In these cases treated with LCP, calcar supporting screws were used routinely. Additionally, rotator cuff suturing technique was performed in all cases regardless of using LCP or conventional plates. The indications for osteosynthesis with plating in the present study were displaced or unstable surgical neck fractures, displaced reconstructible three- and four-part fractures. All of these cases were treated by several trauma surgeons with rich experience in the treatment of humeral fractures.

**Figure 2:**
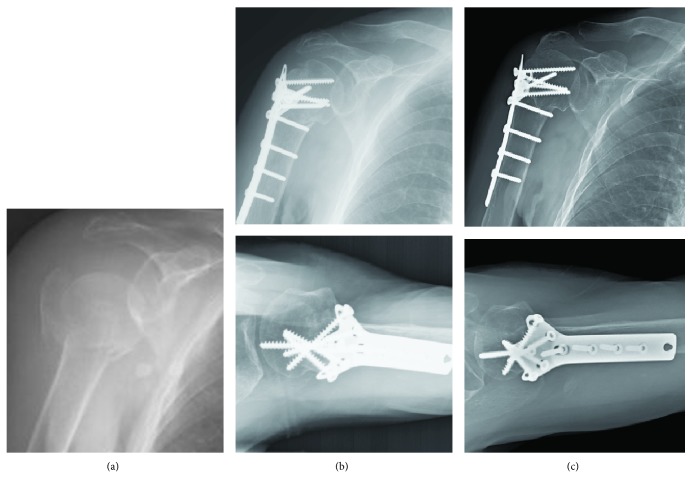
(a) A 71-year-old female patient sustained right proximal humerus fracture AO/OTA 11-B1 with an intact metaphyseal extension and intact medial hinge; (b) open reduction and internal fixation with cloverleaf plate in a mild varus position; (c) external rotation A-P view and axillary view in postoperative 12 months. Solid bone union with acceptable head-shaft angle and no avascular necrosis of the head was noted.

**Figure 3:**
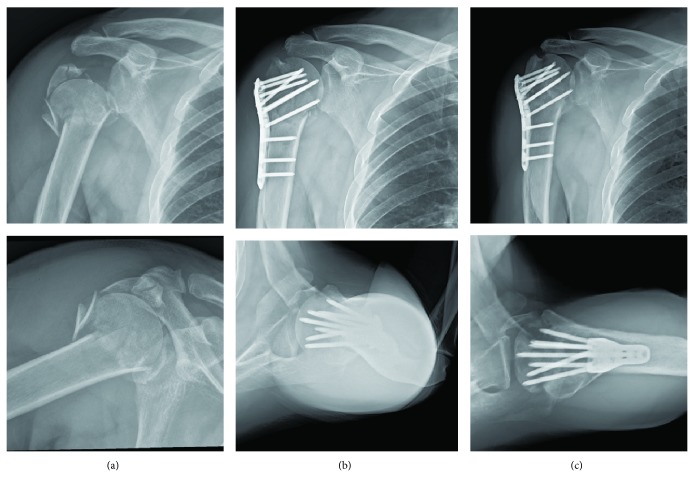
(a) A 51-year-old male patient sustained right proximal humerus fracture AO/OTA 11-B2 with disrupted medial calcar extension and medial hinge sparing; (b) open reduction and internal fixation with PHILOS plating. Anatomical reduction and good strut screw position were achieved; (c) external rotation A-P view and axillary view in postoperative 14 months. Early stages of collapse with less spherical humeral head and impending screw perforation.

**Figure 4:**
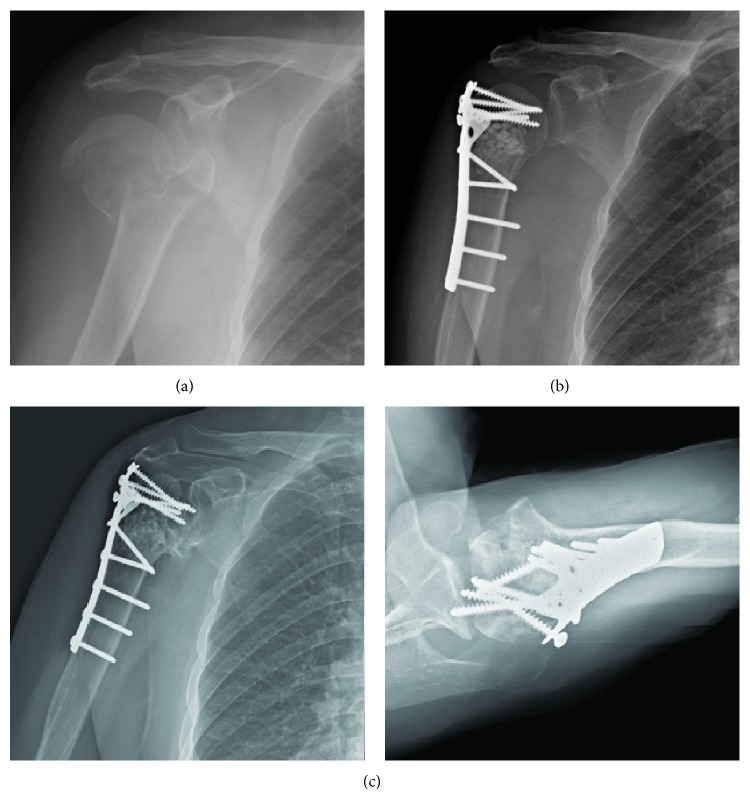
(a) A 69-female patient sustained right proximal humerus fracture AO/OTA 11-A3 with disrupted medial calcar extension and medial hinge with severe shaft medialization; (b) open reduction and internal fixation with cloverleaf plate. Good restoration of head shaft angle and medial hinge; (c) external rotation A-P view and axillary view in postoperative 15 months. Stage IV osteonecrosis with severe head collapse and multiple screws perforation were seen.

To clarify the effects of MCS on radiographic outcomes of plate fixation for PHFx, we classified the enrolled subjects into two groups for further analyses: fractures with intact medial calcar (group 1, *n* = 36) (Figure 2); fractures with disrupted medial calcar (group 2, *n* = 53) (Figures 3 and 4). The information from this study may be valuable in improving plate fixation for PHFx, thereby reducing the complications such as loss of fixation or osteonecrosis of the humeral head.

### 2.2. Definitions

#### 2.2.1. Intact and Disrupted Medial Calcar

Intact medial calcar was defined as the length of the posteromedial metaphyseal extension ≥8 mm on the 20° external rotation anteroposterior radiograph. Those with the posteromedial metaphyseal extension <8 mm were considered as disrupted medial calcar according to the radiographic criteria of Hertel et al. [5].

#### 2.2.2. Nonunion

We defined nonunion as those without evidence of fracture healing, which was determined by a combination of painless palpation of the shoulder and radiographic evidence of bridging bone on plain films within 6 months after injury.

#### 2.2.3. Varus Collapse

It was defined as the varus angulation change of neck-shaft angle >20° on the 20° external rotation anteroposterior view of immediately postoperative radiograph, using the contralateral uninjured shoulder as a reference. The neck-shaft angle, with an average value of 40–45° in normal population, was determined by the angle subtended by the central intramedullary axis of the humeral shaft and the base of the articular segment [6].

#### 2.2.4. Screw Penetration

Acute and late-onset screw perforation of the humeral head was assessed with serial radiographs (20° external rotation anteroposterior and axillary views). Screw penetration was defined as those with >3 mm protrusion through the subchondral bone and those who underwent elective screw removal owning to mechanical symptoms and 1 to 3 mm of screw protrusion through the bone [7].

#### 2.2.5. Redisplacement

Redisplacement was defined as the observed change of variance between the humeral head height and the upper margin of plate on serial follow-up radiographs [8].

#### 2.2.6. Osteonecrosis

Osteonecrosis was defined as those with radiographic evidence of subchondral bone collapse and/or deformity of the humeral head according to the Cruess classification system [9].

#### 2.2.7. Conversion Arthroplasty

Conversion arthroplasty was defined as those with conversion of previous plate fixation for PHFx to total or partial shoulder arthroplasty.

We summarized the collected data at the time of study enrollment, which included demographic data of the patients, fracture classifications, comorbidities, the presence of MCS deficiency, surgical treatments, and radiographic outcomes. We analyzed these patients in a 2-step approach. First, they were compared between patients with and without disruption of medial calcar of PHFx to determine the causal relationship between the disruption of medial calcar and radiographic outcomes. As there was a significant positive causality between the disruption of medial calcar and the occurrence of osteonecrosis of the humeral head in this study, we further classified the patients into two groups according to the presence of osteonecrosis to explore the prognostic factors predicting treatment failure. Second, the risk associations between patient characteristics and radiographic outcomes were analyzed to determine prognostic factors.

### 2.3. Statistical Analysis

A chi-square test or a Fisher's exact test was used for analyzing categorical data. For numerical data, an independent *t*-test was utilized for between-group comparisons. Univariate and multivariate analyses by using a logistic regression model were performed to detect the predicting factors having a significant relationship with poor radiographic outcomes. Statistical significance was defined as *P* < 0.05. All statistics were two-sided and performed using SPSS software (version 17.0. SPSS Inc., Chicago, IL, USA).

### 2.4. Ethic Statement

The data were analyzed after approval by the ethic committee (Institutional Review Board) of the Chang Gung Memorial Hospital in Taiwan. We did not obtain informed consent from the patient due to a statement of this committee that analyzing patient data retrospectively requires no informed consent.

### 2.5. Source of Funding

No external funding was received in support of this study.

## 3. Results

Baseline data on these patients with disrupted or intact medial calcar are shown in Table 1. Age, gender, lesion side, concomitant shoulder dislocation, timely operation, use of locked plate, and selected comorbidities did not differ between the two groups of patients. Regarding the outcome analysis, there were no significant differences in nonunion (*P* = 0.078), screw penetration (*P* = 0.644), and conversion arthroplasty (*P* = 0.269) between the groups. The incidence of osteonecrosis of the humeral head in patients after PHFx surgery was 8.3% and 32.1% (*P* = 0.008), with a significant trend toward a higher rate of osteonecrosis of the humeral head in PHFx having disrupted medial calcar. The incidence of redisplacement after fixation between groups was 30.6% and 50.9% (*P* = 0.050) (Table 2).

There were 20 patients with osteonecrosis of the humeral head and 69 without osteonecrosis of the humeral head. Baseline data on these patients are shown in Table 3. Age, gender, lesion side, concomitant shoulder dislocation, timely operation, and use of locked plate did not differ between the two groups of patients.  Table 4 shows the results of univariate and multivariate analyses of the potential risk factors for an osteonecrosis of the humeral head in patients after PHFx surgery. Using a logistic regression model, varus collapse (OR = 6.75, *P* = 0.010), disrupted medial calcar (OR = 5.16, *P* = 0.038), diabetes mellitus (OR = 6.45, *P* = 0.010), chronic kidney disease (OR = 5.31, *P* = 0.019), and chronic liver disease (OR = 5.56, *P* = 0.027) were all identified as significant predictors for an osteonecrosis of the humeral head in patients after PHFx surgery (Table 4).  Table 5 shows the results of univariate and multivariate analyses of the potential risk factors for redisplacement of fracture reduction in patients after PHFx surgery. Using a logistic regression model, varus collapse (OR = 52.6, *P* = 0.000) and use of locked plating (OR = 0.08, *P* = 0.002) were both identified as significant predictors for redisplacement of reduction in patients after PHFx surgery (Table 5).

20 patients treated with plating osteosynthesis had poor outcome with osteonecrosis of the humeral head. The mean time to osteonecrosis since osteosynthesis was 13.3 months postoperatively. Three patients following plating osteosynthesis had poor outcome with osteonecrosis of the humeral head or loss of fixation and were conversed to prosthetic hemi-arthroplasty.

## 4. Discussion

The present study revealed that there was a trend toward an increased rate of poor radiographic outcomes after plate fixation for PHFx in patients with disrupted medial calcar. Among the selected radiographic outcomes, osteonecrosis of the humeral head and redisplacement of fracture reduction were the two radiographic outcomes which had significant positive casual relationships with disrupted medial calcar. The incidence of osteonecrosis of the humeral head after PHFx surgery in group 1 and group 2 patients was 8.3% and 32.1%, respectively (*P* = 0.008). We, therefore, recommend that the disrupted medial calcar is a predictor of poor stability and vascularity of PHFx and that stability and vascularity should be checked meticulously and repaired accurately before, during, and after surgical treatment.

The two components of MCS are the length of the posteromedial metaphyseal extension and the integrity of the medial hinge combined providing stability and vascularity of the humeral head in PHFx [2–5]. Open reduction and conventional/locked plate fixation remain one of the most popular surgical procedures for treating PHFx owing to that it may restore the anatomy of proximal humerus [1]. However, substantial rates of complications, including nonunion, screw penetration, loss of reduction, and osteonecrosis of humeral head, have been reported in the literature [7, 10–16]. A possible explanation is the MCS deficiency. Ponce et al. [4] showed in their cadaveric biomechanical study that high degrees of MCS deficiency decreased the mean load and the mean energy to failure by 48% and 44%, respectively. Using the calcar screw fixation to restore the MCS, they observed that it could increase the mean load and the mean energy to failure by 31% and 44%, respectively [4]. Hertel et al. [5] studied perfusion of the humeral head in a series of patients with a PHFx and found that the degrees of MCS deficiency were the most relevant predictors of ischemia. Moreover, Gerber et al. [1] reported a high rate (up to 35%) of osteonecrosis of the humeral head without fixation failure developed after PHFx surgery in their 34 consecutive case series. In the present study, the results support the view that the disruption of MCS is associated with high rates of poor radiographic outcomes, particularly the osteonecrosis of humeral head. We, therefore, highlight that the disrupted MCS should be repaired or reconstructed through the refinement of technology and technique focusing on calcar restoration, both mechanically and biologically.

The secondary endpoint of our study was to explore the risk factors predicting treatment failure (i.e., the occurrence of osteonecrosis of the humeral head and redisplacement of fracture reduction post PHFx surgery). Both the fracture geometry (e.g., disrupted medial calcar) and the specific comorbidity (e.g., diabetes mellitus, chronic kidney disease, and chronic liver disease) contributed to the development of osteonecrosis after plate fixation for PHFx. Compared to those without osteonecrosis, patients with osteonecrosis were observed to have a higher rate of the occurrence of inadequate reduction with immediate postoperative varus angulated neck-shaft angle (55.0% versus 26.1%, *P* = 0.015). Recently, Zhang et al. [17] indicated a significant increase in loss of fixation for PHFx with disrupted MCS and less immediate postoperative neck-shaft angle. Our results further demonstrated that there was a risk association between MCS and the development of osteonecrosis of the humeral, with an increased OR of 5.16 and 6.75 in disruption of MCS and varus collapse by multivariate analysis, respectively (*P* = 0.038 and 0.010). For both varus collapse and osteonecrosis of the humeral head developed post-PHFx surgery, it is usually associated with pain, limited shoulder motion, and thus unsatisfactory clinical outcomes [18, 19]. The solution to these problems should be calcar restoration with reestablishment of the MCS during PHFx surgery.

Comorbidities such as diabetes mellitus, chronic kidney disease, and chronic liver disease were all observed to be significant risk factors for developing osteonecrosis of the humeral head in adult patients after PHFx surgery, with OR greater than 5.3 (all *P*-values ≤ 0.027). Surprisingly, little information in the literature is available on the risk association between these comorbidities and the development of postfracture osteonecrosis of the humeral head [14]. Bastian and Hertel [20] reported in their case series study that 8 out of 10 initially ischemic humeral heads did not develop osteonecrosis, indicating that revascularization may indeed occur. However, the reason for advancing osteonecrosis in some of the initially perfused or ischemic heads still remains unclear [10, 11, 18–20]. Although our results suggested that specific comorbidities may play a role in the development of osteonecrosis of the humeral head, further studies are required to conclude a causal association between them.

Our study has several limitations. First, this is a retrospective cohort study harboring all the potential drawbacks implicit in such a study design. Second, the number of patients included during the study period was relatively small, and thus the study may have lacked power to detect the statistical differences in all radiographic outcomes among subsets of patients. Finally, this investigation is lacking in clinical data, including subjective and objective analyses such as bone mineral density, range of motion, muscle power, and functional score. However, the present study was designed to focus and concentrate on the relationship between the MCS deficiency and the poor radiographic outcomes in adult patients with complex PHFx after plate fixation. In the future, additional studies are necessary to determine the effects of MCS on clinical outcomes of PHFx after surgery.

## 5. Conclusions

Our results confirm that the MCS plays a key role in plate fixation for complex PHFx and that there was a trend toward a high rate of poor radiographic outcomes, particularly osteonecrosis of the humeral head and redisplacement of fracture reduction, in the patients having fractures with disrupted calcar and/or lost hinge. We highlight the importance of meticulous check and accurate repair of the MCS deficiency before, during, and after PHFx surgery. With regard to the development of postfracture osteonecrosis of the humeral head, deficient medial calcar, inadequate reduction and comorbid conditions such as diabetes mellitus, chronic kidney, and liver diseases were all recognized as significant risk factors. To clarify and conclude the causality between them, additional studies will be needed to investigate the effects of calcar restoration and comorbid conditions on the reestablishment and maintenance of stability and vascularity of PHFx in patients with MCS deficiency.

## Figures and Tables

**Table 1:** Characteristics among 2-group patients with proximal humerus fracture.

Variables	Group 1	Group 2	*P* value^†^
	(*n* = 36)	(*n* = 53)	
Age, mean years (SD^a^)	66.8 (10.0)	66.3 (11.6)	0.815^‡^
Female gender	28 (77.8)	43 (81.1)	0.699
Right side lesion	16 (44.4)	24 (45.3)	0.935
AO^b^/OTA^c^ type			—
A	22 (61.1)	15 (28.3)	
B	13 (36.1)	27 (50.9)	
C	1 (2.8)	11 (20.8)	
Neer type			—
2 parts	22 (61.1)	16 (30.2)	
3 parts	13 (36.1)	25 (47.2)	
4 parts	1 (2.8)	12 (22.6)	
Shoulder dislocation	4 (11.1)	4 (7.5)	0.710
Timely operation^d^	10 (27.8)	21 (39.6)	0.250
Locked plating	15 (41.7)	22 (41.5)	0.988
Follow-up, mean months (SD)	26.4 (12.8)	27.4 (13.4)	0.729^‡^
Comorbidity			
Diabetes mellitus	12 (33.3)	16 (30.1)	0.754
Arterial hypertension	17 (47.2)	29 (54.7)	0.487
Coronary heart disease	4 (11.1)	4 (7.5)	0.710
Stroke	3 (8.3)	3 (5.7)	0.683
Chronic kidney disease	9 (25.0)	15 (28.3)	0.730
Chronic liver disease^*^	3 (8.3)	14 (26.4)	0.033

Note: data are number (%) of lesions, unless otherwise indicated.

^†^Pearson chi-square test, unless otherwise stated; ^‡^independent *t-*test.

^a^SD: standard deviation; ^b^AO: arbeitsgemeinschaft für osteosynthesefragen; ^c^OTA: orthopaedic trauma association; ^d^timely operation: operation performed <8 hours after fracture.

^*^Statistical significance (*P* < 0.05).

**Table 2:** Subgroup outcome analysis.

Variables	Group 1	Group 2	*P* value^†^
	(*n* = 36)	(*n* = 53)	
Nonunion	0 (0.0)	5 (9.4)	0.078
Screw penetration	2 (5.6)	4 (7.5)	0.644
Redisplacement^*^	**11 (30.6)**	**27 (50.9)**	**0.050**
Osteonecrosis^*^	**3 (8.3)**	**17 (32.1)**	**0.008**
Conversion arthroplasty	0 (0.0)	3 (5.7)	0.269

Note: data are number (%) of lesions, unless otherwise indicated.

^†^Pearson chi-square test, unless otherwise stated.

^*^Statistical significance (*P* < 0.05).

**Table 3:** Characteristics between patients with osteonecrosis and those without osteonecrosis.

Variables	Patients w/ON^a^	Patients w/o ON	*P* value^†^
	(*n* = 20)	(*n* = 69)	
Age, mean years (SD^b^)	68.4 (10.2)	66.0 (10.5)	0.362^‡^
Female gender	16 (80.0)	55 (79.7)	1
Right side lesion	11 (55.0)	29 (42.0)	0.305
AO^c^/OTA^d^ type			—
A	4 (20.0)	33 (47.8)	
B	9 (45.0)	31 (44.9)	
C	7 (35.0)	5 (7.2)	
Neer type			—
2 parts	4 (20.0)	34 (49.3)	
3 parts	8 (40.0)	30 (43.5)	
4 parts	8 (40.0)	5 (7.2)	
Shoulder dislocation	3 (15.0)	5 (7.2)	0.372
Timely operation^e^	10 (50.0)	21 (30.4)	0.106
Locked plating	8 (40.0)	29 (29.4)	0.871
Varus collapse^f,∗^	**11 (55.0)**	**18 (26.1)**	**0.015**
Comorbidity			
Diabetes mellitus^*^	**12 (60.0)**	**16 (23.2)**	**0.002**
Arterial hypertension	11 (55.0)	35 (50.7)	0.736
Coronary heart disease	2 (10.0)	6 (8.7)	1
Stroke	1 (5.0)	5 (7.2)	1
Chronic kidney disease^*^	**11 (55.0)**	**13 (18.8)**	**0.001**
Chronic liver disease^*^	**8 (40.0)**	**9 (13.0)**	**0.019**

Note: data are number (%) of lesions, unless otherwise indicated.

^†^Pearson chi-square test, unless otherwise stated; ^‡^independent *t-*test.

^a^ON: osteonecrosis; ^b^SD: standard deviation; ^c^AO: arbeitsgemeinschaft für osteosynthesefragen; ^d^OTA: orthopaedic trauma association; ^e^timely operation: operation performed <8 hours after fracture; ^f^varus collapse: >20° varus on immediate postoperative follow-up radiographs.

^*^Statistical significance (*P* < 0.05).

**Table 4:** Prognostic factors of osteonecrosis in patients with proximal humerus fracture.

Variables	Univariate		Multivariate^†^	
	OR^a^ (95% CI^b^)	*P* value	OR (95% CI^b^)	*P* value
Age	1.02	0.359		
Female gender	1.02	0.977		
Shoulder dislocation	2.26	0.296	—	—
Timely operation^c^	0.44	0.111	—	—
Locked plating	0.92	0.871	—	—
Varus collapse^d,∗^	3.46	0.018	**6.75**	**0.010**
Disrupted calcar^*^	5.19	0.014	**5.16**	**0.038**
Diabetes mellitus^*^	4.97	0.003	**6.45**	**0.010**
Chronic kidney disease^*^	5.27	0.002	**5.31**	**0.019**
Chronic liver disease^*^	4.44	0.010	**5.56**	**0.027**

^†^Multivariate: including all variables with univariate *P* value < 0.05.

^a^OR: odds ratio; ^b^CI: confidence interval; ^c^timely operation: operation performed <8 hours after fracture; ^d^varus collapse: >20° varus on immediate postoperative follow-up radiographs.

^*^Statistical significance (*P* < 0.05).

**Table 5:** Prognostic factors of redisplacement of fracture reduction in patients with proximal humerus fracture.

Variables	Univariate		Multivariate^†^	
	OR^a^ (95% CI^b^)	*P* value	OR (95% CI^b^)	*P* value
Age	1.03	0.174	—	—
Female gender	1.64	0.371	—	—
Shoulder dislocation	0.17	0.105	—	—
Timely operation^c^	0.38	0.034	0.56	0.381
Locked plating^*^	**0.16**	**0.000**	**0.08**	**0.002**
Varus collapse^d,∗^	**34.67**	**0.000**	**52.6**	**0.000**
Disrupted calcar	2.36	0.059	3.10	0.096
Diabetes mellitus	0.82	0.660	—	—
Chronic kidney disease	1.89	0.187	—	—
Chronic liver disease	1.24	0.686	—	—

^†^Multivariate: including age, sex, and all variables with univariate *P* value < 0.05.

^a^OR: odds ratio; ^b^CI: confidence interval; ^c^timely operation: operation performed <8 hours after fracture; ^d^varus collapse: >20° varus on immediate postoperative follow-up radiographs.

^*^Statistical significance (*P* < 0.05).
